# Investigation of the effects of the CFTR potentiator ivacaftor on human P-glycoprotein (ABCB1)

**DOI:** 10.1038/s41598-017-17773-5

**Published:** 2017-12-13

**Authors:** Swathi Lingam, Nopnithi Thonghin, Robert C. Ford

**Affiliations:** Faculty of Biology, Medicine and Health, The University of Manchester, Michael Smith building, Oxford road, Manchester, M13 9PL UK

## Abstract

Ivacaftor is a potentiator of the CFTR chloride channel and is in worldwide clinical use for the chronic treatment of cystic fibrosis in patients. There is evidence that the bioavailability of ivacaftor in the body may be influenced by the multi-drug exporter P-glycoprotein. Here we have employed purified and reconstituted P-glycoprotein to study its interaction with ivacaftor as well as the ability of the drug to compete with a known transported substrate of the protein. We find that ivacaftor stimulates the ATPase activity of the purified protein and can compete with the transport of the fluorescent substrate Hoechst 33342. These findings lead us to conclude that ivacaftor is very likely an efficiently transported substrate of P-glycoprotein. Evidence for state-dependent binding of ivacaftor was obtained using a fluorescent, cysteine-reactive reporter dye. The quiescent, nucleotide-free state in the P-glycoprotein transport cycle appears to bind ivacaftor strongly.

## Introduction

P- glycoprotein (P-gp) is an ABC transporter that is typically expressed in the apical membranes of brain capillary endothelial cells (in the blood brain barrier)^1^, pancreas, kidney, liver^2^ and placenta^3^. It was first identified in a Chinese Hamster ovary cell line that was found to be resistant to colchicine^4^. P-gp transport activity is known to cause reduced therapeutic drug bioavailability in several cancers and also in chronic conditions such as epilepsy^5,6^. In the latter example, lower bioavailability of the drugs is caused by reduced uptake in the intestine and at the blood-brain barrier^7^. In cancer cells, P-gp causes multidrug resistance by actively exporting therapeutic drugs, hence reducing their efficacy^5^. Due to the ability of this protein to transport a large variety of unrelated compounds, newly developed drugs are tested as potential allocrites of P-gp. One such drug that was recently tested was the cystic fibrosis transmembrane conductance regulator (CFTR) potentiator ivacaftor or VX-770^8^.

Cystic fibrosis (CF) is an inherited, life – threatening disease that affects multiple organs such as the lungs and intestines. CF leads to the accumulation of mucus and consequently the blockage of ducts, which leads to secondary infections due to accumulation of bacteria^9^. Mutations in the CFTR gene are responsible for CF^10^. CFTR is expressed in the apical side of the plasma membrane and is involved in the transport of chloride ions across the membrane^11^. One of the most common CFTR mutations is G551D, which causes defective channel gating. This and several related channel gating mutations may be potentially treated with Ivacaftor or VX- 770 that works by increasing channel opening, hence improving channel gating^12,13^.

*In vitro* studies in human liver microsomes showed that ivacaftor could inhibit CYP2C8, CYP2C9 and CYP3A. In Caco- 2 cells, ivacaftor was able to inhibit P-gp^8^. Based on these studies, an *in vivo* clinical drug- drug interaction (DDI) study was carried out to test the effect of ivacaftor on substrates of CYP3A, CYP2C8, CYP2D6 and P-gp. This study showed that ivacaftor appeared to weakly inhibit CYP3A and P-gp (marginally increased bioavailability of midazolam and digoxin respectively)^8^.

However, these systems are extremely complex and do not provide a clear picture on how P-gp interacts with ivacaftor. Previous studies on P-gp have utilised membrane vesicles to study the effect of drugs on the protein^5^. In our study, we expressed wild – type human P-gp (WT hP-gp) in a *Saccharomyces cerevisiae* system. Human P-gp was purified from detergent solubilised yeast membranes and the purified, lipid reconstituted protein system was used to study the effect of ivacaftor on hP-gp.

## Materials and Methods

### Materials

Yeast growth media was obtained from Formedium (Hunstanton, Norfolk, UK). The reagents for protein expression, buffer components, CPM dye, verapamil, nicardipine and disodium ATP were from Sigma-Aldrich (Dorset, UK). N- dodecyl- β-D- maltoside (DDM) was from Merck Chemicals (Nottingham, UK). The protein purification columns were from GE healthcare (Buckinghamshire, UK). Brain polar lipids were from Avanti Polar Lipids (Alabaster, Alabama, USA). Hoechst 33342 was from Thermo Fisher scientific (Waltham, Massachusetts, USA). Ivacaftor was from Vertex pharmaceuticals (Boston, Massachusetts, USA). Tariquidar was from BioVision (Cambridge, UK).

### Expression of WT hP-gp in *Saccharomyces cerevisiae*

The WT hP-gp DNA used in this study was kindly provided by Dr. Ian Kerr (University of Nottingham)^14^. The DNA was isolated from its host vector using PCR. *BamH1/Xma1* restriction sites were also introduced at the 5′ and 3′ ends of the DNA and used to clone it into a modified p424GAL1 vector^15^. This vector has a GAL1 promoter and C- terminal GFP and 10X His tags for protein purification and visualisation. This construct was transformed into FGY217 *S. cerevisiae* as described previously^15^.

### Protein purification

WT hP-gp was expressed and microsomes were prepared as described previously^16^. These were diluted to 2 mg/mL in solubilisation buffer (50 mM Tris pH 8.0, 30% glycerol, 50 mM NaCl and 2% DDM). P-gp was purified from the soluble material by nickel affinity chromatography followed by size exclusion chromatography as described previously^16,17^, with the following modifications: Only one HisTrap column was used. The bound protein was sequentially washed with purification buffer containing 20 mM and 80 mM imidazole and the protein was eluted into buffer containing 400 mM imidazole and a Superdex 200 column was used for size – exclusion chromatography. The purified material was concentrated to 2 mg/mL using a 100 kDa cut – off concentrator (Vivaspin), flash frozen in liquid nitrogen and stored at −80 °C.

### CPM thermal stability assay

CPM (7-Diethylamino-3- (4′-Maleimidylphenyl)-4-Methylcoumarin) was used to monitor the thermal unfolding of WT hP-gp^18^. The assay was performed using a fluorimeter with a 266 nm excitation source (Unchained Labs). The initial temperature was set to 15 °C and the final temperature was set to 90 °C. Fluorescence emission spectra were recorded between 266 nm and 700 nm every 2 °C at a heating rate of 0.8 °C per min. The 473 nm laser was turned off.

WT hP-gp with and without 1 μM ivacaftor, 1 μM tariquidar, 1 μM nicardipine and 5 μM verapamil was diluted to 0.3 μg in 10 μl purification buffer. The samples were prepared as described previously^19^. All reactions were set up in triplicate and the thermal unfolding of two separate protein preparations was estimated.

The emission data recorded from 480 nm to 500 nm was integrated to estimate the CPM fluorescence of the samples. The mid- point unfolding temperature was estimated as described previously^20^.

### Lipid reconstitution

To remove the protein from its detergent environment, it was reconstituted into lipids. The lipids were prepared and the reconstitution was carried out as previously described^16^, except that in this study WT hP-gp was reconstituted into brain polar lipids supplemented with cholesterol (4:1 ratio by mass, respectively).

### ATPase assay

The basal and ivacaftor/verapamil stimulated ATPase activity of WT hP-gp was estimated using a modified Chifflet assay^21,22^. Reconstituted membranes containing 0.25 μg protein were used per reaction. The ATPase activity was measured as described previously^16^ with and without 1 μM ivacaftor or 5 μM verapamil. The reactions were set up in triplicate.

### Hoechst 33342 transport assay

Hoechst 33342 transport by P-gp in the presence and absence of inhibitors was measured as described previously [23]. The only difference was that 0.25 μg of proteoliposomes was used per assay, rather than 1 μg.

### Statistical analysis

Unpaired two- tailed Student’s T- test (GraphPad Prism 7) was utilised. At least 3 independent repeats were considered for statistical analysis. The difference was considered to be significant if the P-value < 0.05. To calculate the IC50 value of ivacaftor, tariquidar and nicardipine, the initial rate of Hoechst 33342 transport (%) was plotted against drug concentration (GraphPad Prism 7). Non- linear regression was used to estimate the IC50 value.

### Data availability

The datasets generated and analysed during this study that are not included in the published article are available from the corresponding author upon reasonable request.

## Results

### Purification of WT hP-gp

**Figure 1:**
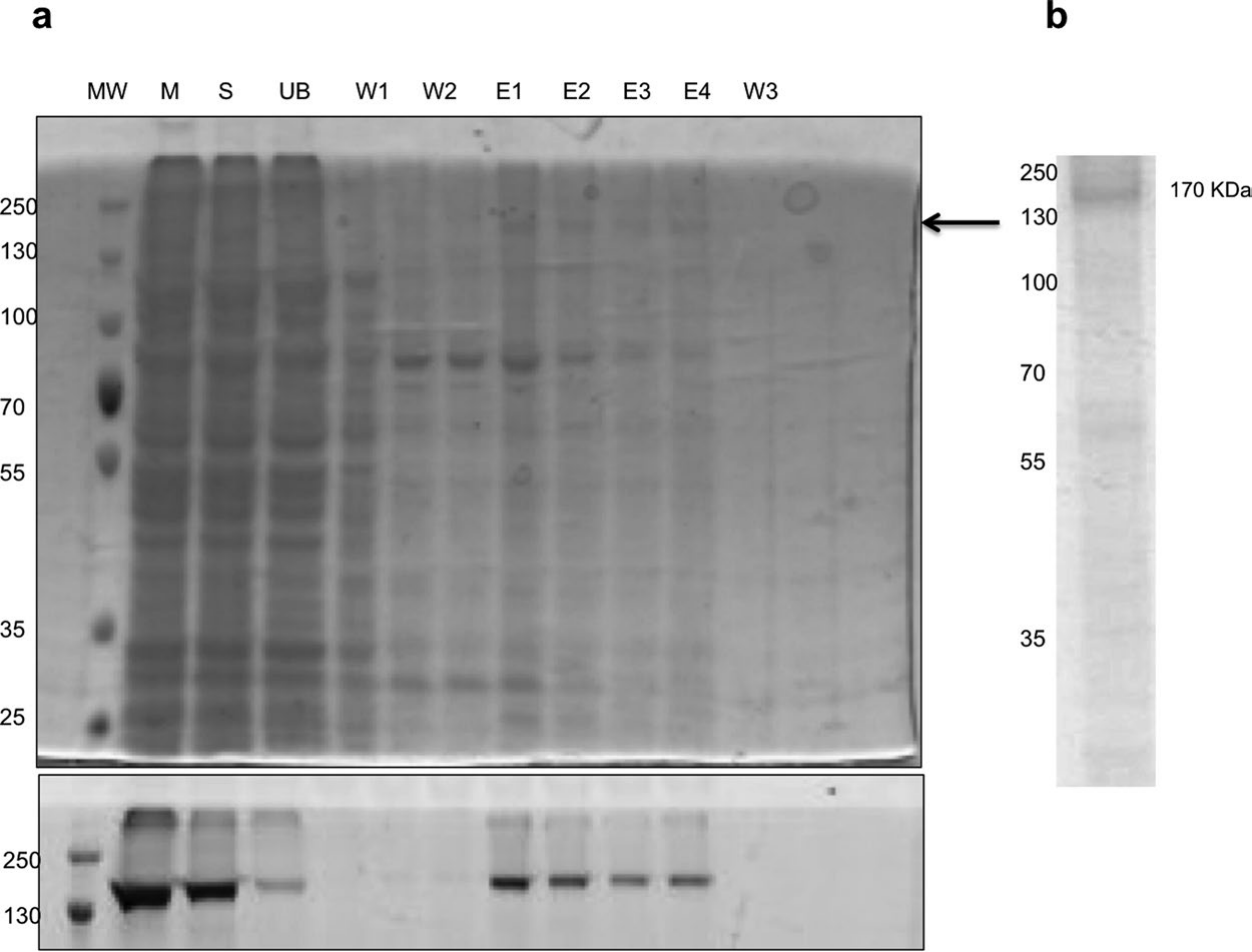
Purification of WT hP-gp. (**a**) Coomassie-stained gel showing purification fractions. Lanes are indicated as MW-molecular weight marker, M-microsomes, S- soluble fraction, UB unbound to the HisTrap column; W1, W2 are 20 mM and 80 mM imidazole wash fractions respectively. The four 400 mM imidazole elution fractions are E1-E4 and a final 1 M imidazole wash fraction is W3. WT hP-gp is indicated by the arrow. The lower panel shows the fluorescence scan of the unstained gel with the GFP tag fluorescence being detected. The original gel was modified to show only WT hP-gp. The complete gel is presented in Supplementary Figure 1. (**b**) Coomassie- stained gel of the main fraction after size- exclusion chromatography. The original gel was cropped to present one fraction. The SEC purified protein fractions were concentrated to 2 mg/ mL and were ~ 90% pure as estimated by densitometry of the gel. The complete gel is presented in Supplementary Figure 1.

WT hP-gp was purified from the DDM solubilised microsomes with two chromatographic steps, the first being nickel- affinity chromatography (Fig. 1a, lanes 6–9) followed by size- exclusion chromatography (Fig. 1b). The final fractions were pooled and concentrated to a concentration of 2 mg/mL. This material was judged to be ~ 90% pure (Fig. 1b). The faint band visible at ~70 kDa in Fig. 1b was likely to be a C – terminal fragment of WT hP-gp based on the GFP fluorescence scan which showed a fluorescent band at this relative mass.

### Ivacaftor binds to WT hP-gp and affects its thermal stability

**Figure 2:**
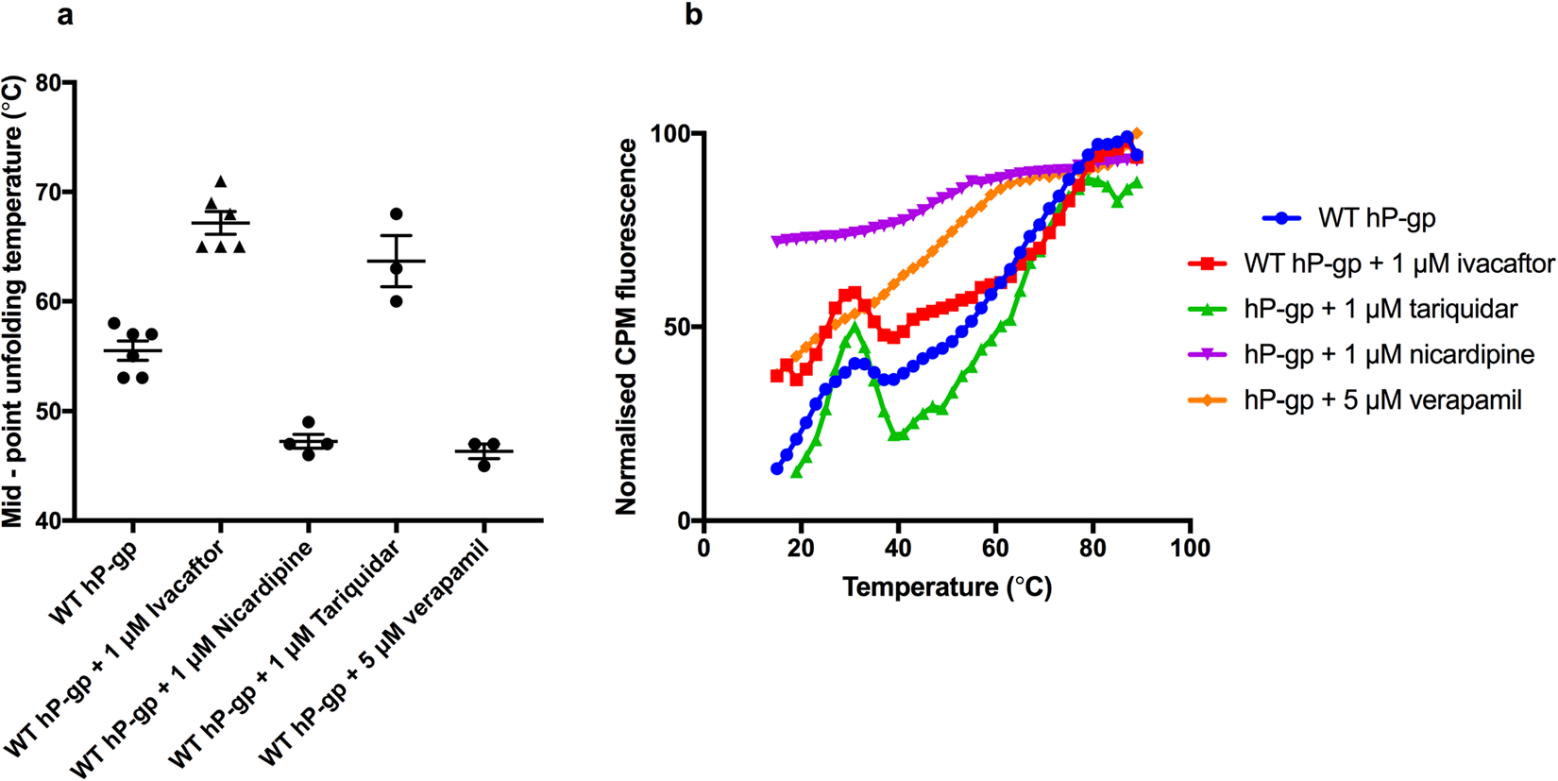
Ivacaftor stabilises WT hP-gp. The thermal stability of WT hP-gp was probed with and without 1 μM ivacaftor, nicardipine, tariquidar and 5 μM verapamil using two independently purified batches of protein. (**a**) Summary of the mean mid- point temperatures of unfolding of WT hP-gp with and without allocrites. The protein on its own had an apparent mid- point unfolding temperature of 55.5 ± 0.9 °C (n = 6). In the presence of ivacaftor, this shifted to 67.2 ± 1.1 °C (n = 6). The difference was statistically significant (P- value < 0.0001). With tariquidar the apparent mid- point unfolding temperature was 63.7 ± 2.3 °C (P- value = 0.0047) (n = 3), with nicardipine it was 47.3 ± 0.6 °C (P- value = 0.0001) (n = 4) and with verapamil it was 46.3 ± 0.7 °C. (**b**) Full thermal unfolding curves with and without ivacaftor, tariquidar, nicardipine and verapamil. The addition of ivacaftor and tariquidar shifted the curve to the right, indicating an apparent stabilisation of the protein. Nicardipine and verapamil on the other hand appeared to destabilise the protein and did not display a minor transition at about 30 °C. The data in panel A represent the mean ± SEM for the midpoint of the unfolding transition. The data in panel B represent the mean normalized values, but error bars are omitted for the sake of clarity.

The thermal stability of the purified protein in DDM was probed using a CPM assay (Fig. 2a,b). CPM is a thiol- reactive dye that fluoresces when it binds to cysteine residues. There was some initial labelling due to CPM binding to exposed cysteines present in the native state. As the protein was heated, unfolding caused buried cysteine residues to become surface exposed as reported by the formation of cysteine- CPM adducts, and an increase in CPM fluorescence^18^.

We hypothesised that if ivacaftor could bind to WT hP-gp, it could alter the thermal stability of the protein. To this end, the thermal stability of WT hP-gp was tested in the absence and presence of 1 μM ivacaftor. Tariquidar, nicardipine and verapamil were also tested since they are known modulators of P-gp that bind to the protein^23–25^. The apparent mid- point unfolding temperature of WT hP-gp determined by the CPM assay was about 56 °C. This is similar to the reported melting temperature of lipid-reconstituted murine P-gp^26^. This was increased to 67 °C in the presence of 1 μM ivacaftor (Fig. 2), implying that ivacaftor was binding to WT hP-gp and caused a stabilisation of the protein. Tariquidar also appeared to significantly stabilise the protein in this assay (T_m_ = 64 °C). On the other hand, two other known P-gp substrates, verapamil and nicardipine, appeared to destabilise the protein in this assay (T_m_ = 46 & 47 °C respectively). There is evidence that drugs can bind to membrane proteins while causing an apparent destabilisation^20^.

As far as we are aware, this is the first time that the effects of allocrites on the thermal stability of P-gp have been reported. WT hP-gp without drugs and in the presence of ivacaftor and tariquidar showed a minor transition around 30 °C with an initial increase in CPM fluorescence followed by thermal quenching before the main cooperative unfolding event was initiated. This lower temperature transition was absent in the thermal unfolding profiles of WT hP-gp with nicardipine and verapamil. It is possible that WT hP-gp on its own and with ivacaftor and tariquidar has a native state where partially buried cysteine(s) are not exposed until the temperature rises above 15 °C. In contrast, with verapamil and nicardipine these cysteine(s) appear to be accessible to the CPM dye even at 4 °C, probably reflecting a global structural difference in the presence of these drugs.

### Ivacaftor stimulates the ATPase activity of WT hP-gp

**Figure 3:**
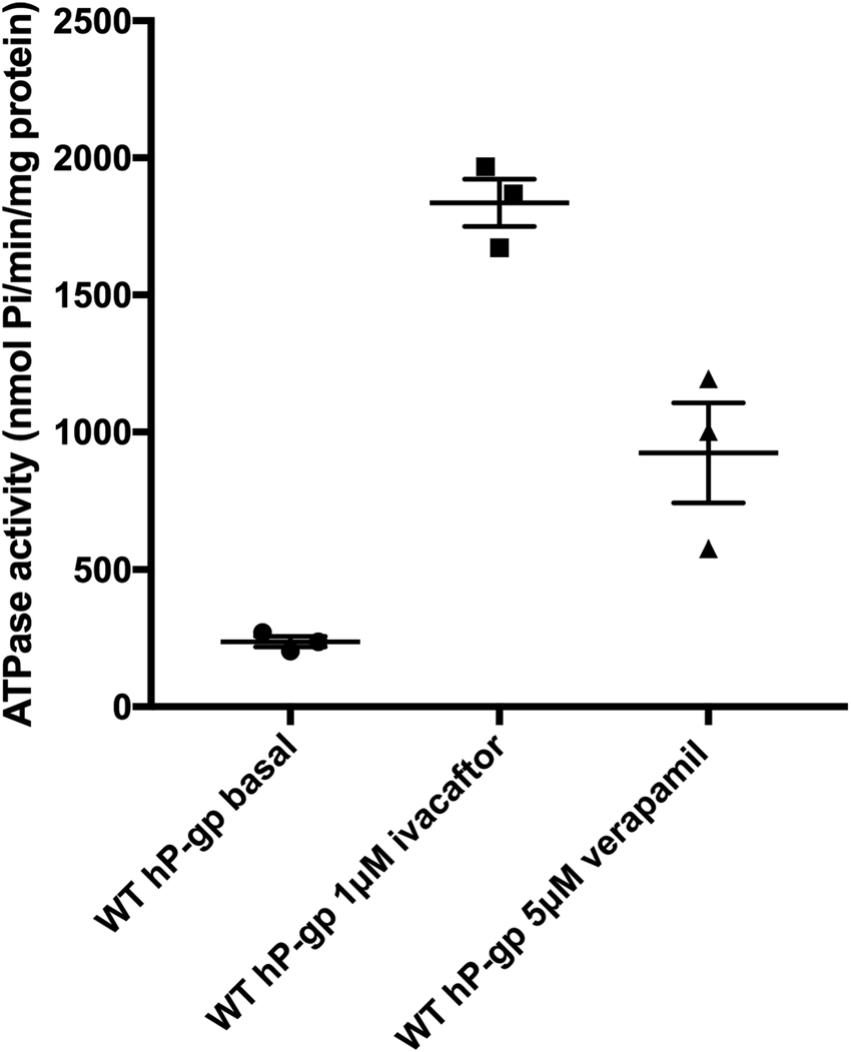
Ivacaftor stimulates the ATPase activity of WT hP-gp. The basal ATPase rate was 237 ± 20 nmol Pi/ minute/mg of protein. This was stimulated nearly 8 fold with 1 μM ivacaftor (1836 ± 87 nmol Pi/ minute/mg of protein). The difference in ATPase activity was statistically significant (P- value < 0.0001). The data are represented as mean ± SEM of three independent repeats. For comparison, the ATPase activity was stimulated to 924 ± 182 nmol Pi/ minute/mg of protein by the addition of the well-characterised substrate verapamil (P- value = 0.0201).

Purified WT hP-gp was reconstituted into brain polar lipids and its ATPase activity was tested in the absence and presence of 1 μM ivacaftor. Without stimulation, WT hP-gp had a basal ATPase activity of 237 ± 20 nmol Pi/minute/mg protein. This was stimulated 7.7 fold to 1836 ± 87 nmol Pi/minute/mg protein with ivacaftor (Fig. 3). As a positive control, a known P-gp substrate was also tested. Verapamil stimulated the ATPase activity to 924 ± 182 nmol Pi/minute/mg protein). Ivacaftor stimulated ATPase activity was therefore significantly higher than the verapamil (5 μM) stimulated activity (P- value = 0.01).

### Ivacaftor modulates the transport of Hoechst 33342

**Figure 4:**
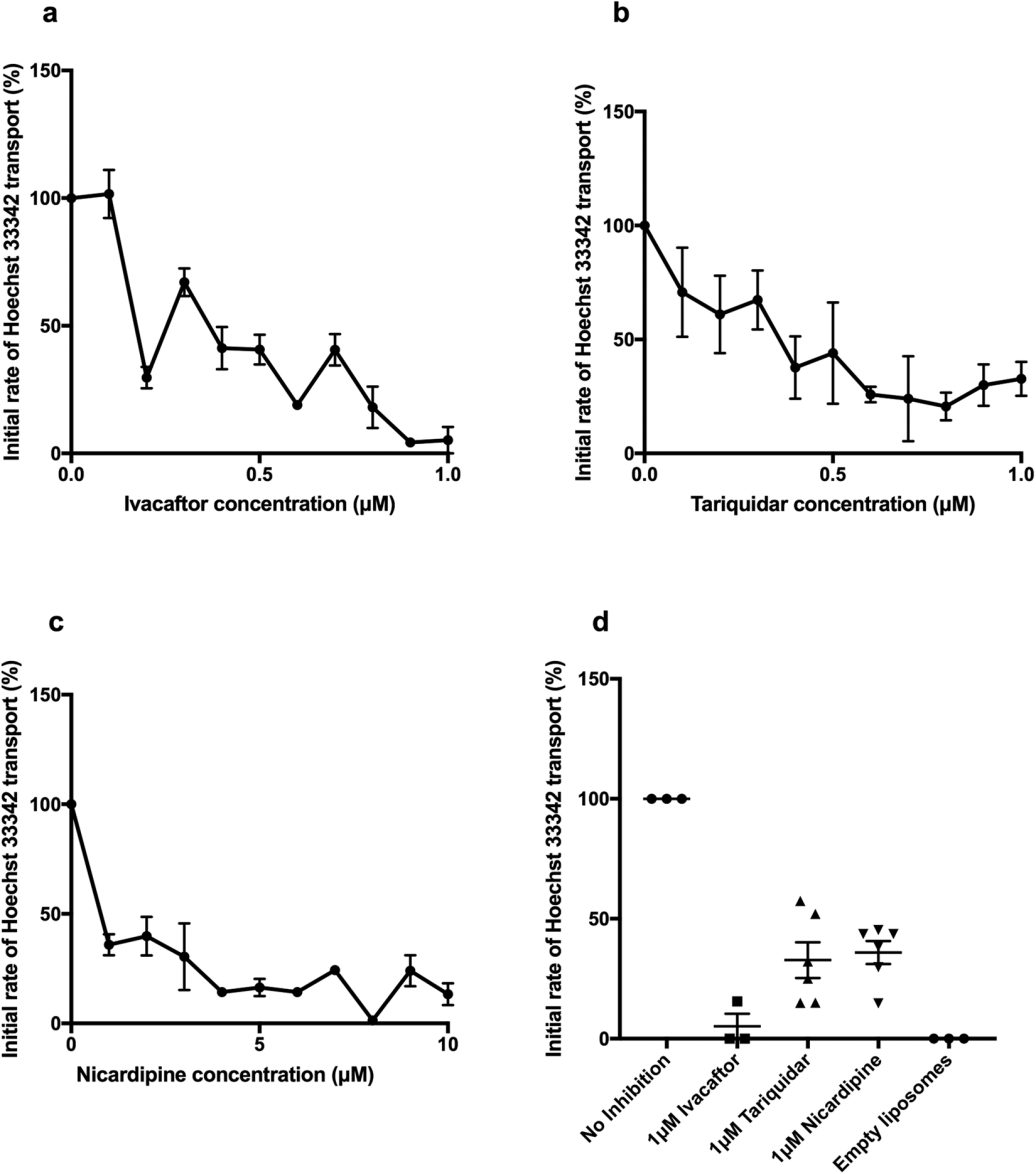
Ivacaftor inhibits the transport of Hoechst 33342. (**a**) Ivacaftor dose- response. The IC50 value was 0.27 ± 0.05 μM. There are some points where the error bars are too small to be visible. (**b**,**c**) Similar dose- response curves for well characterised modulators tariquidar and nicardipine were estimated in the same way. The IC50 value of tariquidar was 0.33 ± 0.06 μM. Nicardipine had an IC50 value of 0.82 ± 0.12 μM. (**d**) Comparison of the ability of lipid-reconstituted WT hP-gp to transport Hoechst 33342 at 1 μM concentrations of the three compounds. Ivacaftor had the largest effect at this concentration.

Hoechst 33342 is a known substrate of P-gp. It is a useful substrate to test transport activity since it fluoresces strongly in a lipid environment, but its fluorescence is quenched in an aqueous environment. Hence in a lipid reconstituted system when ATP is added, inside- out WT hP-gp will transport Hoechst 33342 into the aqueous lumen of proteoliposomes, leading to a time- dependent quenching of fluorescence [23]. The rate of fluorescence change in the first 10 seconds after the addition of ATP was employed to determine the initial rates of Hoechst 33342 transport. In all cases the fluorescence reached a new steady – state within a few minutes where Hoechst 33342 retrograde diffusion matched P-gp-induced efflux; as discussed in^23^. Empty liposomes with and without ivacaftor were used as controls. Overall, ivacaftor was found to reduce the initial rate of transport of Hoechst 33342 transport by about 95% (Fig. 4a) in a dose-dependent manner, whilst tariquidar and nicardipine reduced Hoechst 33342 transport to about ~20% of control values (Fig. 4a–c). Dose- response curves (Fig. 4a–c) generated IC50 values for ivacaftor (0.27 ± 0.05 μM), tariquidar (0.33 ± 0.06 μM) and nicardipine (0.82 ± 0.12 μM). Figure 4d summarises all the Hoechst 33342 transport data collected for the three different drugs with multiple repeat assays at a concentration of 1 μM drug. These data suggest that there is a significant difference between ivacaftor and tariquidar, despite their having similar IC50 values estimated from the dose-response curves.

## Discussion

Previous *in vivo* studies suggested that the CFTR potentiator ivacaftor was either a weak inhibitor of hP-gp or a competitive substrate as it mildly increased the bioavailability of digoxin in patients. In this study, we were interested in studying the interaction of ivacaftor with purified hP-gp in both detergent solubilised form as well as in a reconstituted proteoliposome system.

The changes in thermal stability of WT hP-gp with and without ivacaftor imply that the drug can bind directly to the detergent-solubilised protein. Presumably the drug is binding to the inward-facing state since in these experiments there is no ATP present. Structures of murine P-gp show that the ATP-free state, both with and without drug are in an inward – facing conformation^27,28^ with the nucleotide-binding domains separated. WT hP-gp has seven cysteine residues of which six are conserved in murine P-gp (mP-gp). Comparison of the hP-gp sequence with the mP-gp crystal structures shows that there are potentially three buried cysteine residues and four surface – exposed cysteine residues, two of which lie in the NBD-NBD interface^27^. In addition, in our construct there will be one buried and one exposed Cys residue in the GFP tag. Hence we would expect to observe about half of the maximal CPM fluorescence in the native unfolded state at 15 °C. In contrast, for WT hP-gp the CPM signal starts at only 15% of the maximal signal (Fig. 3b, blue trace). These data therefore imply that several P-gp Cys residues that appear to be surface- located in the murine P-gp structures may be constrained by the dynamics of their local environment, preventing CPM adduct formation at 15 °C. In agreement with this model, a small degree of kinetic energy appears to induce CPM adduct formation for these partially constrained Cys residues, with a transition observed giving a small peak centred at about 30 °C which corresponds to about 40% of the maximal CPM signal (Fig. 3b). We hypothesise that such a low temperature transition is unlikely to arise from domain unfolding, but rather may be due to the dynamics of the ~60 residue long linker region that connects NBD1 with TMD2. Although this region is absent from the murine P-gp structures because of partial disorder, there is evidence that it may intercalate between the NBDs^29^. Hence the linker region could hinder CPM labelling of the Cys residues at the NBD interface at 15 °C but a relatively small input of kinetic energy could dissociate the linker from this region.

Purified mP-gp was reported to have a mid – point melting temperature of approximately 42 °C in the detergent DDM as estimated using differential scanning calorimetry (DSC) and circular dichroism (CD)^26^. This is lower than the main melting transition reported here. This discrepancy may be due to intrinsic differences in the mP-gp and hP-gp orthologues or due to differences in their preparation protocols. Moreover the CPM assay may be subject to some hysteresis because of the CPM adduct formation kinetics.

The ATPase activity of WT hP-gp was measured after reconstitution into brain polar lipids supplemented with cholesterol. These lipids were used since this combination was reported to be close to the lipid composition of the human blood brain barrier^30^. There is some evidence that the composition of the lipid bilayer can influence the partitioning of drugs and their interaction with P-gp^31,32^. With this lipid combination, the basal ATPase activity of roughly 200 nmol Pi/minute/mg protein could be stimulated to 1800 nmol Pi/minute/mg protein with 1 μM ivacaftor. The drug – stimulated ATPase activity of hP-gp has been extensively studied, with most studies employing either verapamil or nicardipine^25,33,34^. The stimulated ATPase activity in this study was comparable to that of verapamil stimulated mP-gp, hP-gp and Chinese hamster P-gp^34,35^ and nicardipine stimulated hP-gp^14^.

The transport of the P-gp substrate Hoechst 33342 + ATP has previously been used to test the transport capabilities of lipid reconstituted Chinese hamster and human P-gp^31,35^. Such transport assays have also been used to study the effect of competing substrates and modulators on the transport of Hoechst 33342 and tetramethyl rhodamine (TMR)^23,36^. In our system, ivacaftor could significantly reduce the transport of Hoechst 33342. It appeared to be more potent than known P-gp modulators tariquidar and nicardipine, which were tested as positive controls for the experiment. Ivacaftor had an IC50 value of 0.27 ± 0.05 μM, which was similar to the IC50 value determined in a Caco- 2 cell system (0.17 μM)^8^. The overall maximal reduction in Hoechst 33342 transport by ivacaftor is higher than observed for other allocrites such as tariquidar, nicardipine (this study), verapamil and cyclosporine A^23,36^. Although it is possible that ivacaftor can reduce the Hoechst 33342 transport by inhibitory action, its stimulation of the ATPase activity implies it is more likely to be a competitive substrate.

In conclusion, this study shows that the CFTR potentiator ivacaftor is probably a transported allocrite of hP-gp. From the data presented in this paper, it is likely that ivacaftor binds to and causes an apparent stabilisation of purified hP-gp. Ivacaftor could also stimulate the ATPase activity of brain polar lipid reconstituted hP-gp 7 – fold. This behaviour is similar to that of several hP-gp modulators and substrates.

## Electronic supplementary material

Supplementary Information
