# Supplementary material for: Investigation of the effects of the CFTR potentiator ivacaftor on human P-glycoprotein (ABCB1)
Source: Sci Rep. 2017 Dec 13;7:17481. doi: 10.1038/s41598-017-17773-5 (PMC5727471; doi:10.1038/s41598-017-17773-5)

Investigation of the effects of the CFTR potentiator ivacaftor on human P-glycoprotein (ABCB1)

Authors: Swathi Lingam, Nopnithi Thonghin, Robert. C. Ford*
Affiliations: Faculty of Biology, Medicine and Health, The University of Manchester, Michael Smith building, Oxford road, Manchester M13 9PL, UK.

Supplementary figure 1: Complete gels of WT hP-gp purification. The gel on the left is the fluorescence scan of the gel. WT hP-gp in each fraction is visible between the 250kDa and 130kDa molecular weight markers. The gel on the right is the complete Coomassie stained version of the same gel. The gel below is the complete Coomassie stained gel of the size exclusion chromatography fractions. Fractions 1-5 were concentrated to 2 mg/mL.

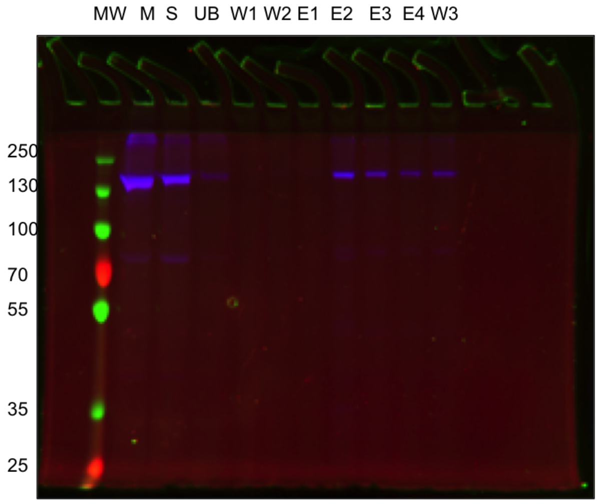

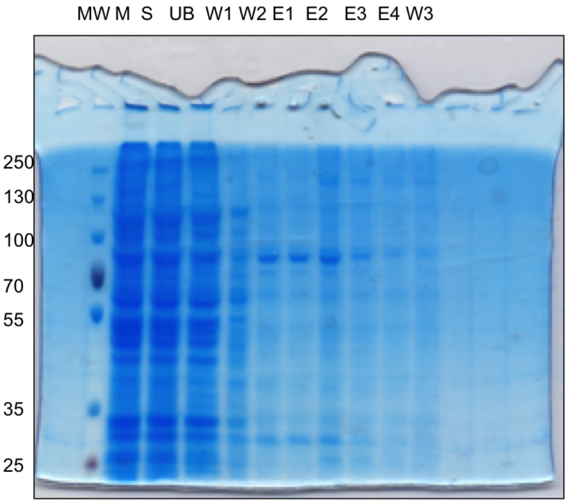

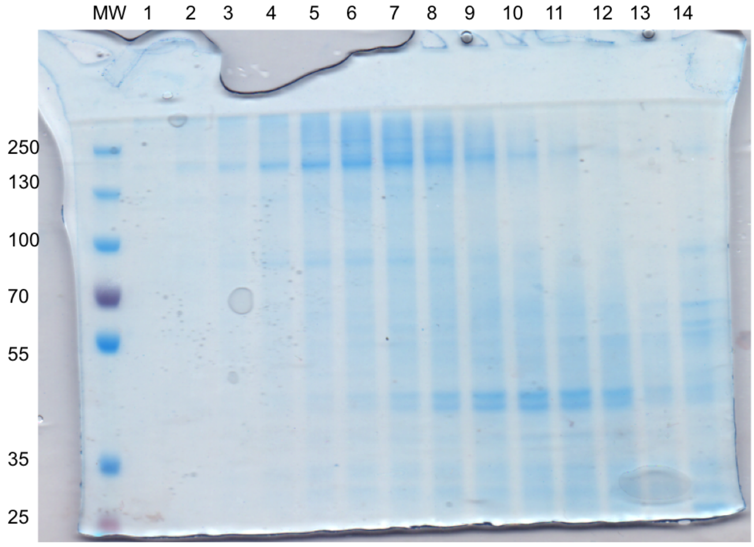

Supplementary figure 2: Hoechst 33342 transport measured in empty liposomes. There is no Hoechst 33342 transport by empty liposomes as determined by the lack of decrease in Hoechst 33342 fluorescence upon the addition of ATP (at 0 minutes).

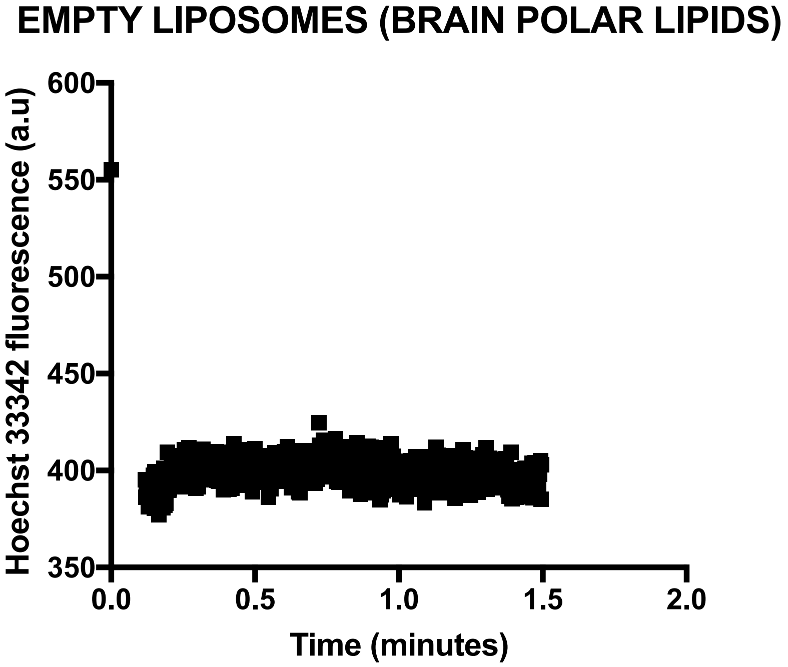

Supplement: Supplementary file 1 — Supplementary Information [file 41598_2017_17773_MOESM1_ESM.pdf]
